# LoCoLotive: In silico mining for low‐copy nuclear loci based on target capture probe sets and arbitrary reference genomes

**DOI:** 10.1002/aps3.11535

**Published:** 2023-07-28

**Authors:** Ulrich Lautenschlager, Agnes Scheunert

**Affiliations:** ^1^ Evolutionary and Systematic Botany Group, Institute of Plant Sciences University of Regensburg Universitätsstraße 31 Regensburg D‐93053 Germany; ^2^ Genomics Core Facility of the SNSB Menzinger Straße 67 D‐80638 Munich Germany

**Keywords:** low‐copy loci, marker mining, nuclear marker, probe kit, single‐copy loci, target capture, target enrichment

## Abstract

**Premise:**

Universal target enrichment probe kits are used to circumvent the individual identification of loci suitable for phylogenetic studies in a given taxon. Under certain circumstances, however, target capture can be inefficient and costly, and lower numbers of marker loci may be sufficient. We therefore propose a computational pipeline that enables the easy identification of a subset of promising candidate loci for a taxon of interest.

**Methods and Results:**

Target sequences used for probe design are filtered based on an assembled reference genome, resulting in presumably intron‐containing single‐copy loci as present in the reference taxon. The applicability of the proposed approach is demonstrated based on two probe kits (universal and family‐specific) in combination with several publicly available reference genomes.

**Conclusions:**

Guided by commercial probe kits, LoCoLotive enables fast and cost‐efficient marker mining. Its accuracy mainly depends on the quality of the reference genome and its relatedness to the taxa under study.

In molecular systematics, the de novo identification of nuclear loci suitable for phylogenetic analyses in a given set of taxa can be a challenging task because the molecular markers have to satisfy several requirements. First, for primers or probes to bind effectively, conserved, non‐repetitive regions are required for amplification via PCR or for enrichment via target capture using pre‐designed probes. Second, appropriate loci need to feature some degree of variability, meaning they should carry a phylogenetic signal. Third, for many analyses, loci are expected to be single‐copy because paralogs may be subject to different genealogies. The first two (contrary) prerequisites can usually be met by targeting intronic regions that are flanked by more conserved exonic regions. The third aspect is of particular importance in the case of plants, as Panchy et al. (2016) estimated that, on average, about 65% of land plant genes are paralogous. All three requirements can be easily met, but only if an assembled reference genome is available.

Today, researchers greatly benefit from sequencing whole collections of conserved nuclear loci using next‐generation sequencing and target enrichment based on pre‐designed probes (e.g., Lemmon and Lemmon, 2013; Zimmer and Wen, 2015; Yu et al., 2018; Andermann et al., 2020). As opposed to classical PCR‐based amplification, target capture via the hybridization of probes (which typically target exonic regions) allows the simultaneous extraction of multiple marker loci while avoiding artifacts typical of PCR. Moreover, the availability of so‐called universal probe kits (e.g., Angiosperms353; Johnson et al., 2019), which are applicable to various distantly related genera, potentially eliminates the effort of identifying suitable marker loci for each individual taxon of interest; however, this approach can be inefficient in lineages with large genome sizes due to a reduced number of on‐target reads. For example, in a study involving a sample with a C‐value of 16.25 pg, Jones et al. (2019) reported the corresponding proportion of on‐target reads in sequenced data to be as low as 1.92%. Large genome sizes are particularly common in polyploid taxa (see below). Target capture is also of limited utility in cases where single‐copy loci may be required, such as when trying to elucidate the evolutionary history of (allo)polyploids. Unfortunately, the genes targeted by commercially available probe kits cannot necessarily be expected to be single‐copy, especially when utilized for taxa other than the ones originally used for the probe design. Furthermore, several approaches to phylogenetic network reconstruction in the presence of allopolyploids (e.g., Jones et al., 2013; Oberprieler et al., 2017; Lautenschlager et al., 2020) do not scale well with the number of input loci, meaning that, in practice, it may be adequate to use a comparatively small number of appropriate markers for analysis. Here, we describe LoCoLotive, a novel pipeline that uses the target sequences of available probe kits (i.e., the exonic sequences used to design the shorter capture probes) for in silico mining of suitable single‐ or low‐copy loci in a given taxonomic group. The pipeline, mainly consisting of shell scripts, is easy to use and was designed to quickly identify promising intron‐containing loci at essentially no cost for the user, assuming appropriate input data are already available. This approach therefore represents a straightforward analysis solution when working with non‐model taxa for which a related reference genome is available, even if they lack taxon‐specific genomic information, which is a common situation in many plant groups. LoCoLotive can be used in initial efforts, before considering more costly or time‐consuming alternatives, such as the de novo identification of markers, target capture, or whole‐genome sequencing.

## METHODS AND RESULTS

### Workflow and implementation

As a first step, a nucleotide BLAST (MegaBLAST; Zhang et al., 2000; Morgulis et al., 2008; Camacho et al., 2009) is performed to identify similar regions (“matches”) between target sequences and the reference genome, subject to a certain *E*‐value threshold. This is done locally, therefore a new BLAST database is created beforehand if needed. The resulting BLAST hits are then successively filtered according to the following criteria.

First, target sequences with fewer than two BLAST hits are discarded. As intronic rather than purely exonic regions are of interest, only target sequences matching different parts of the reference genome are considered because they are much more likely to contain an intron. For the sake of efficiency, only the total number of hits per target sequence (rather than location) is considered in this step; location is addressed in the following steps. Second, target sequences with matches on different reference sequences (i.e., chromosomes, scaffolds, or contigs, depending on the assembly level) are discarded. Third, target sequences with matches on both strands of the same reference sequence are discarded. Fourth, target sequences are discarded for which a particular part, exceeding a certain threshold length, maps to different regions on the same strand of the same reference sequence. Repeated mapping suggests unwanted duplication of the locus covered by the target sequence, and even partial duplication may counteract eventual primer design.

Note that steps 2–4 aim to exclude different types of duplications. In doing so, we take a restrictive approach by accepting the loss of some suitable loci rather than keeping potentially problematic ones. For each of the remaining target sequences, its reference‐matching parts, along with the reference region covering all corresponding BLAST hits, are then realigned using MAFFT (Katoh et al., 2002; Katoh and Standley, 2013). If an annotation comprising exon information is available for the genome assembly used, the intronic regions are inferred and highlighted in uppercase in the multiple sequence alignments (MSAs) produced by MAFFT. Here, by default, only strictly intronic regions are considered, meaning that parts of an intron that also belong to an exon (e.g., on the opposite strand) are ignored as they may not have the desired degree of variability. The MSAs represent the primary output of the proposed pipeline; a tabular output is also provided, which summarizes several characteristics such as the alignment length, number of and distance between BLAST hits, and the number of enclosed intronic base pairs. Additional output files display the regions of a target sequence involved in matches to the reference genome. Depending on the input files, multiple target sequences may map to overlapping or even identical regions of the reference genome. In this case, LoCoLotive searches for groups of overlapping candidate loci, indicates the group membership of each locus in the tabular summary, and provides further output files such as groupwise MSAs and a groupwise summary to facilitate subsequent screening.

The whole pipeline, which mainly consists of shell scripts designed for Linux operating systems, can be run using a convenient main script written in Python 3 (Van Rossum and Drake, 2009). Alternatively, the shell scripts can be modified and run manually to satisfy more specialized requirements. The main script stores its outputs in a nested directory hierarchy, which is particularly useful when the pipeline is run multiple times using different input files or parameter settings. Certain possibly time‐consuming steps, such as the BLAST search, are only performed if their results are not yet present in the directory hierarchy, or if the user explicitly chooses to recompute them via the ‐r/‐‐run_all option. In addition to the input files (i.e., the target sequences, the reference genome, and an optional genome annotation), two numeric parameters can be specified by the user: the *E*‐value threshold used for the BLAST search and a threshold length for duplicated fragments (MC_LENGTH), which regulates the behavior of the fourth filter criterion. A lower MC_LENGTH parameter results in stricter filtering of the loci, as it is more likely that short fragments would be duplicated. The default value of 15 may seem relatively low, but is motivated by the required specificity of primers that might be built based on the outcome. While a high *E*‐value threshold may lead to false positives and thus erroneously assumed duplications, applying an overly low value may miss relevant matches. The default value of 10^–5^ aims to account for this trade‐off. As we are more concerned about the single‐copy state of proposed candidate loci rather than capturing all potentially suitable loci, higher values tend to be safer than lower ones.

Because the pipeline depends on several tools and programming languages, including BEDOPS (Neph et al., 2012), BEDTools (Quinlan and Hall, 2010), BLAST, FASTX‐Toolkit (http://hannonlab.cshl.edu/fastx_toolkit), gawk (https://www.gnu.org/software/gawk/), GenomeTools (Gremme et al., 2013), MAFFT, bash (https://www.gnu.org/software/bash/), Python 3, and R (R Core Team, 2022), future versions of them might lead to compatibility issues. We provide two solutions to this problem: a Docker (Merkel, 2014) image and a Conda (https://docs.conda.io/projects/conda/) environment. Both contain all required dependencies and can be used as tested environments to run the pipeline. The Docker image was used to perform the sample analyses below.

### Example analyses

To prove its applicability across different clades and genome sizes, LoCoLotive was first used to identify suitable marker loci in *Aegilops* L., *Artemisia* L., *Cinnamomum* Schaeff., *Coffea* L., *Cynara* L., *Pyrus* L., *Setaria* P. Beauv., *Triticum* L., and related genera based on publicly available target sequences from the Angiosperms353 probe set, using only sequences belonging to the same order as the reference genome. All of the references used, along with assembly levels, sizes, and taxa, are listed in Table 1. The analyses were performed using the default parameter settings, and genome annotations were considered if available. As *Triticum aestivum* L. (2*n* = 6*x*) and *Coffea arabica* L. (2*n* = 4*x*) are allopolyploids, we ran the pipeline based only on single subgenomes, and included scaffolds without subgenome assignment to avoid unwanted duplications as much as possible. It should be noted that LoCoLotive does not distinguish between homoeologs and paralogs; therefore, using complete polyploid assemblies comprising multiple subgenomes may result in very few (if any) candidate loci.

**Table 1:** Summary of LoCoLotive runs based on target sequences of the Angiosperms353 probe set and various reference genomes. Each run was performed using default parameter settings. For each reference taxon, only target sequences belonging to the same order were used. Values marked with an asterisk are distorted by outliers, while the values in parentheses were obtained after outlier removal.

Target sequences		Reference genome				Number of candidate loci	Avg. alignment length (bp)	Number of groups	Avg. alignment length (bp), groupwise
Order	Number	Taxon	GenBank assembly accession	Assembly level	Size (bp)				
Asterales	187	*Artemisia annua*	*GCA_003112345.1*	Scaffold	1,792,856,094	24	3729	21	3545
		*Cynara cardunculus* var. *scolymus*	*GCA_001531365.2*	Chromosome	724,962,400	122	4177	113	397,193*(4191)
Gentianales	64	*Coffea arabica*	*GCA_003713225.1*	Chromosome	1,094,291,418	8	2822	7	2900
		*Coffea arabica*, Subgenome c			603,167,519	31	3068	30	3103
		*Coffea arabica*, Subgenome e			595,493,427	35	2768	33	2805
		*Coffea canephora*	*GCA_900059795.1*	Chromosome	568,611,505	42	3062	40	3108
		*Coffea eugenioides*	*GCA_003713205.1*	Chromosome	699,903,961	36	2639	34	2668
Laurales	96	*Cinnamomum micranthum* f. *kanehirae*	*GCA_003546025.1*	Scaffold	730,416,403	56	9911	n.a.	n.a.
Poales	676	*Aegilops tauschii* subsp. *strangulata*	*GCA_002575655.2*	Chromosome	4,218,064,899	394	2862	223	3110
		*Setaria italica*	*GCA_000263155.2*	Chromosome	405,732,883	440	2545	241	19,391*(2661)
		*Triticum aestivum*	*GCA_018294505.1*	Chromosome	14,566,502,436	0	n.a.	n.a.	n.a.
		*Triticum aestivum*, Subgenome A			5,310,640,027	382	3132	217	3386
		*Triticum aestivum*, Subgenome B			5,586,571,631	346	3189	198	3461
		*Triticum aestivum*, Subgenome D			4,335,614,492	380	2823	216	3092
Rosales	69	*Pyrus betulifolia*	*GCA_007844245.1*	Chromosome	532,747,033	14	1848	n.a.	n.a.

All results based on the Angiosperms353 kit are summarized in Table 1. The number of available target sequences for the order Poales in Angiosperms353 is considerably higher than for the other tested orders, which is reflected by the fact that LoCoLotive suggested the highest numbers of loci (max. 440) and disjunct groups of loci (max. 241) for reference taxa belonging to this order. At the other end of the spectrum, only 14 candidate loci could be identified for target sequences from the Rosales, using *Pyrus betulifolia* Bunge as the reference taxon. Within the Asterales, there is a striking difference between the number of loci suggested for *Artemisia annua* L. (24) and *Cynara cardunculus* L. var. *scolymus* (L.) Benth. (122). This is likely to be partly caused by the different assembly levels, as scaffolds (or contigs) are more susceptible than chromosomes to the second filter criterion. Furthermore, a larger reference genome may sometimes comprise more duplications and therefore result in a lower number of candidate loci. However, this is not always the case; for example, despite the *Aegilops tauschii* subsp. *strangulata* (Eig) Tzvelev assembly being approximately 10 times as large as that of *Setaria italica* (L.) P. Beauv., both of these Poaceae references yield similar numbers of candidate loci, with a slight reduction in the former taxon being compensated by longer loci.

As expected, very few candidate loci could be identified when using complete polyploid assemblies. Based on the whole *Coffea arabica* assembly, only eight loci passed the filtering, whereas its subgenomes (31 and 35 loci; 24 targets in common) yielded similar numbers of loci to the closely related diploids *Coffea canephora* Pierre ex A. Froehner (42) and *Coffea eugenioides* S. Moore (36).

Similarly, no loci could be retrieved based on the complete *Triticum aestivum* assembly, while its individual subgenomes (382, 346, and 380 loci; 286 targets in common) yielded almost the same number of loci as *Aegilops tauschii* subsp. *strangulata* (394), which is closely related to subgenome D.

Across the analyses summarized in Table 1, the observed alignment sizes are more consistent than the numbers of loci. The only outlier is *Cinnamomum micranthum* f. *kanehirae* (Hayata) S. S. Ying, with an average alignment length of 9911 bp; the average alignment lengths for the rest of the references ranged from 1848 to 4177 bp.

The results also show a possible problem when dealing with grouped outputs due to overlapping loci. There may occasionally be outlier groups that are orders of magnitude longer than the other groups, which would distort the average length of the groupwise alignments. In *Cynara cardunculus* var. *scolymus*, we find two such groups of 15,649,825 and 28,759,340 bp, respectively, and one of 4,031,781 bp in *Setaria italica*. In both cases, MAFFT failed to compute such huge alignments within the runtime limits imposed by LoCoLotive, which are aimed at avoiding excessive resource usage. By removing these outlier groups, the average lengths of the groupwise alignments decreased from 397,193 bp and 19,391 bp to 4191 bp and 2661 bp for *C. cardunculus* var. *scolymus* and *S. italica*, respectively, which were now similar to related reference taxa. (For the outlier groups, the alignment lengths were previously estimated using the genomic coordinates from BLAST due to the lack of proper MSAs.)

**Figure 1:**
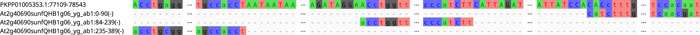
Sample output multiple sequence alignment produced by LoCoLotive. The first sequence represents a fragment of the reference scaffold *PKPP01005353.1*, in which intronic bases are shown as uppercase letters. Below, three parts of the target sequence *At2g40690sunfQHB1g06_yg_ab1* that have been matched to the reference are shown. Here, the target sequence fragments have been reverse‐complemented, as indicated by a minus sign appended to the sequence names. For better illustration, all sequences are shortened using dots as placeholders. In this alignment, which has a total length of 1434 bp, intronic regions largely correspond to the unmatched parts of the reference (see Appendix S1).

For the *Artemisia* and *Cynara* references, we also tested a set of loci introduced for the Compositae family by Mandel et al. (2014). The corresponding target capture probes have been designed on lettuce (*Lactuca sativa* L.), sunflower (*Helianthus annuus* L.), and safflower (*Carthamus tinctorius* L.) based on expressed sequence tags (ESTs), which served as target sequences in the following analyses. To avoid redundancy, for *Artemisia annua* (Anthemideae) and *Cynara cardunculus* var. *scolymus* (Cardueae), only the source ESTs from sunflower (1061 loci) and safflower (475 loci), respectively, were used as input for our pipeline, while using the same reference assemblies as above. Here, we also studied the effects of varying the *E*‐value threshold and the MC_LENGTH parameter. The results are summarized in Table 2. Despite the higher number of target sequences used for *A. annua* than for *C. cardunculus* var. *scolymus*, we still obtained more candidate loci when using the latter as the reference taxon. While the results for *C. cardunculus* var. *scolymus* seem to be similar to the Angiosperms353‐based analysis, candidate loci for *A. annua* are much shorter on average when using the Compositae‐specific set of target sequences. Because ESTs from single taxa were used, no overlapping loci were detected. Within the given parameter settings, the *E*‐value threshold seems to have a greater impact on the results than the MC_LENGTH parameter. A higher *E*‐value threshold increases the number of BLAST hits, sometimes leading to false positives, and therefore increases the number of target sequences (not to be confused with the “subject sequences” of BLAST, sometimes also referred to as “targets”) passing the first filter step. However, this results in a more aggressive filtering due to putative duplications, which explains the decreasing numbers of candidate loci. As expected, lower MC_LENGTH settings also lead to stricter filtering and therefore fewer candidate loci. Overall, however, the pipeline seems to be relatively robust with respect to the specific parameter setting. For both reference taxa, the average alignment length remains almost constant when changing the MC_LENGTH or the *E*‐value threshold. Sample outputs for *A. annua* are shown in Figure 1 and Appendix S1.

**Table 2:** Summary of LoCoLotive runs based on source ESTs of a Compositae‐specific probe set and two reference Compositae genomes. Only target sequences belonging to either sunflower or safflower were used. For both input combinations, multiple runs were performed using different settings for the *E*‐value threshold and the MC_LENGTH parameter.

Target sequences		Reference genome				*E*‐value threshold	MC_LENGTH	Number of candidate loci	Avg. alignment length (bp)
Species	Number	Taxon	GenBank assembly accession	Assembly level	Size (bp)				
*Helianthus annuus* (sunflower)	1061	*Artemisia annua*	*GCA_003112345.1*	Scaffold	1,792,856,094	1e‐10	10	61	1536
						1e‐5	10	61	1495
						1	10	56	1524
						10	10	55	1514
						1e‐10	15	64	1533
						1e‐5	15	64	1473
						1	15	59	1499
						10	15	57	1504
						1e‐10	25	64	1533
						1e‐5	25	65	1456
						1	25	60	1480
						10	25	57	1504
*Carthamus tinctorius* (safflower)	475	*Cynara cardunculus* var. *scolymus*	*GCA_001531365.2*	Chromosome	724,962,400	1e‐10	10	124	4338
						1e‐5	10	113	4381
						1	10	100	4466
						10	10	94	4509
						1e‐10	15	140	4557
						1e‐5	15	127	4481
						1	15	113	4438
						10	15	106	4485
						1e‐10	25	148	4514
						1e‐5	25	133	4487
						1	25	119	4449
						10	25	110	4505

All analyses were performed on a Dell (Round Rock, Texas, USA) Optiplex 7010 desktop PC with an Intel (Santa Clara, California, USA) i5‐3470 CPU and 12 GB RAM, using the supplied Docker image. With the exception of the Poales targets, where LoCoLotive took between 11 and 18 min to finish, and the Asterales/*Cynara* analysis (9 min), all runs finished within approximately 3 min.

## CONCLUSIONS

As demonstrated, the presented pipeline enables the identification of promising putative single‐ or low‐copy loci for a group of interest by analyzing and filtering published target sequences of available target capture probe kits. The resulting subset of loci (or groups of overlapping loci) is considerably reduced and can be easily inspected using the produced alignments and filtered based on the tabular output. Possible considerations for filtering might include the total length of the fragment (depending on whether standard or long‐range PCR is intended), the lengths of the constituent introns, and the general suitability of the intronic sequence regarding amplifiability and phylogenetic reconstructions. For this last consideration, it could be helpful to check for the presence of longer homopolymers or repetitive elements such as microsatellites.

The genomic sequence of the reference, as present in the alignment, then serves as a basis for primer design. Popular primer design tools such as Primer3Plus (Untergasser et al., 2012) take a genomic sequence as input, and the regions to be used for primer design can be easily restricted to the (known) exonic regions. Adhering to general good practice for primer design will increase amplification success, e.g., including a GC clamp at the 3′ end of the primer, keeping the melting temperatures of the two oligos within 5°C of each other, and avoiding primer self‐complementarity and dimerization. The latter two aspects can easily be accounted for using tools such as the Oligonucleotide Properties Calculator (Kibbe, 2007). The chosen primer pairs should be BLASTed against the full reference genome to identify possible secondary binding sites; the Primer‐BLAST software (Ye et al., 2012) can also be used for this.

The applicability and reliability of the proposed approach mainly depend on the availability of a suitable, preferably annotated, reference genome. The more closely the reference is related to the taxa of interest, the higher the chances that a single‐copy locus in the former is single‐copy in the latter. The assembly level of the reference genome may also influence the quality and number of the proposed candidate loci. The latter number is also particularly affected by the set of target sequences used. When working with polyploids, it is usually recommended to use either genomes of diploid relatives or single subgenomes of a polyploid as the reference to avoid discarding loci represented by multiple homoeologs. Of course, when applied to a polyploid, candidate loci identified in this way cannot be expected to be single‐copy sensu stricto, yet paralogs are minimized. Care should also be taken in cases where a genome assembly comprises sequences from multiple alleles as these will likely be treated as duplications.

Marker mining based on a reference genome does not necessarily require input from markers designed on other taxa; however, utilizing available probe kits, whose target sequences are typically already the result of multiple filter steps, concentrates the search on promising regions of the reference genome and is therefore less computationally expensive. While a de novo approach might identify a higher number of suitable loci, LoCoLotive focuses on cases where a limited number of informative single‐ or low‐copy loci is sufficient. The required availability of the target sequences (e.g., ESTs) used for probe design poses some restriction on the probe sets that can be leveraged; however, this limitation may be mitigated by the use of the universal Angiosperms353 kit. Filtering input target sequences for certain lineages may speed up computations, but is not necessarily required, given LoCoLotive's ability to identify and summarize groups of overlapping loci.

While LoCoLotive relies on a standard nucleotide BLAST and the identification of relevant groups of BLAST hits, other tools such as exonerate (Slater and Birney, 2005) provide dedicated methods for aligning exonic sequences to a given genome. However, for removing duplications based on the proposed filter criteria, standard local alignments are sufficient. As the first three filter steps are very fast and the slightly slower fourth step acts on an already reduced set of target sequences, our approach is expected to be faster than performing more sophisticated alignment methods for all target sequences and subsequently removing duplicates. This makes it particularly suitable for exploratory use.

In summary, while techniques like probe‐based target enrichment are generally viewed as a means to enable larger‐scaled analyses, we demonstrate here that such developments may also benefit more traditional, smaller‐scaled approaches for data acquisition.

## AUTHOR CONTRIBUTIONS

U.L. and A.S. conceived the proposed methodology. U.L. implemented the software, carried out the analyses, and wrote a first draft of the manuscript, which was improved with input from A.S.

## Supporting information


**Appendix S1**. Tabular output of LoCoLotive applied to expressed sequence tags (ESTs) from sunflower using *Artemisia* as a reference. The table provides a brief summary for each target sequence that passed all filtering steps. In most cases, reference regions located between consecutive BLAST hits of the same target are also annotated as intronic.

## Data Availability

LoCoLotive's source code and usage information are freely available at https://github.com/AGOberprieler/LoCoLotive. Further usage guidelines are provided in the README file. The complete example analyses can be reproduced using the example_analyses.sh script.
