# Supplementary material for: LoCoLotive: In silico mining for low‐copy nuclear loci based on target capture probe sets and arbitrary reference genomes
Source: Appl Plant Sci. 2023 Jul 28;11(6):e11535. doi: 10.1002/aps3.11535 (PMC10719872; doi:10.1002/aps3.11535)
Supplement: Supplementary file 1 — Appendix S1. Tabular output of LoCoLotive applied to expressed sequence tags (ESTs) from sunflower using Artemisia as a reference. The table provides a brief summary for each target sequence that passed all filtering steps. In most cases, reference regions located between consecutive BLAST hits of the same target are also annotated as intronic. [file APS3-11-e11535-s001.docx]

**Appendix S1**. Tabular output of LoCoLotive applied to expressed sequence tags (ESTs) from sunflower using *Artemisia* as a reference. The table provides a brief summary for each target sequence that passed all filtering steps. In most cases, reference regions located between consecutive BLAST hits of the same target are also annotated as intronic.

| **Target sequence (EST)** | **Alignment length (bp)** | **Number of BLAST hits** | **Distance between BLAST hits (bp)** | **Intronic base pairs between BLAST hits** |
| --- | --- | --- | --- | --- |
| At2g41490sunfQHB39M10_yg_ab1 | 2737 | 7 | 554, 107, 86, 201,  220, 919 | 554, 106, 86, 201,  179, 707 |
| At2g45740sunfQHA6E16_yg_ab1 | 1075 | 6 | 88, 76, 126, 73, 92 | 88, 76, 126, 73, 92 |
| At2g25310sunfQHB27H08_yg_ab1 | 2393 | 5 | 340, 858, 77, 660 | 340, 858, 77, 660 |
| At5g09920sunf32533766 | 2028 | 5 | 76, 551, 213, 790 | 76, 551, 179, 578 |
| At1g09340sunfQHL15L12_yg_ab1 | 1986 | 5 | 761, 59, 437, 189 | 761, 59, 437, 186 |
| At1g72560sunfQHB34M20_yg_ab1 | 1947 | 5 | 562, 67, 651, 100 | 562, 67, 651, 100 |
| At3g15840sunf32531845 | 1011 | 5 | 113, 288, 76, 99 | 113, 288, 76, 81 |
| At5g22360sunfQHB12A10_yg_ab1 | 8970 | 4 | 1129, 1720, 5576 | 0, 1720, 5576 |
| At3g19900sunfQHK16J03_yg_ab1 | 2286 | 4 | 212, 360, 1354 | 212, 360, 1354 |
| At2g31040sunfQHL14B04_yg_ab1 | 2107 | 4 | 429, 1107, 189 | 429, 1107, 189 |
| At2g26210sunfQHF6P03_yg_ab1 | 1428 | 4 | 297, 74, 794 | 297, 51, 739 |
| At5g56290sunfQHB5K04_yg_ab1 | 1097 | 4 | 509, 78, 71 | 509, 78, 71 |
| At3g27340sunf32532122 | 2387 | 3 | 83, 1991 | 83, 1991 |
| At1g77310sunfQHF5L22_yg_ab1 | 1671 | 3 | 123, 1351 | 123, 1348 |
| At1g52230sunf32541355 | 1646 | 3 | 566, 679 | 0, 0 |
| At3g44680sunfQHB42P10_yg_ab1 | 1578 | 3 | 501, 831 | 0, 0 |
| At2g40690sunfQHB1g06_yg_ab1 | 1434 | 3 | 577, 458 | 577, 458 |
| At2g27810sunfQHE14D15_yg_ab1 | 800 | 3 | 91, 82 | 91, 82 |
| At1g06680sunf32531066 | 648 | 3 | 85, 147 | 85, 147 |
| At2g16950sunfQHB11M02_yg_ab1 | 571 | 3 | 255, 83 | 255, 83 |
| At3g19910sunfQHB42M01_yg_ab1 | 484 | 3 | 70, 124 | 69, 124 |
| At3g62810sunf28370960 | 7746 | 2 | 7443 | 7441 |
| At4g00585sunfQHK17L05_yg_ab1 | 4672 | 2 | 4461 | 4461 |
| At3g48680sunfQHB41E18_yg_ab1 | 4629 | 2 | 4165 | 4165 |
| At5g17840sunf32530581 | 4381 | 2 | 4062 | 0 |
| At5g53650sunf32535021 | 3661 | 2 | 3443 | 0 |
| At5g42960sunf32534405 | 2794 | 2 | 2452 | 2452 |
| At5g65220sunfQHM9N11_yg_ab1 | 2227 | 2 | 1939 | 1939 |
| At1g08780sunf32541317 | 1997 | 2 | 1631 | 1631 |
| At1g65040sunfQHG12P13_yg_ab1 | 1646 | 2 | 1338 | 1338 |
| At3g47860sunfQHM18G16_yg_ab1 | 1540 | 2 | 1128 | 1128 |
| At2g22370sunfQHN18H13_yg_ab1 | 1464 | 2 | 1273 | 1272 |
| At1g05910sunfQHG3c10_yg_ab1 | 1296 | 2 | 1016 | 1016 |
| At1g67170sunf32538536 | 1103 | 2 | 868 | 868 |
| At1g75330sunfQHL9C02_yg_ab1 | 987 | 2 | 676 | 676 |
| At4g27500sunfQHA7P21_yg_ab1 | 968 | 2 | 667 | 665 |
| At4g31780sunf28371375 | 940 | 2 | 642 | 642 |
| At2g13360sunfQHB7D19_yg_ab1 | 921 | 2 | 543 | 543 |
| At1g50575sunf32533435 | 870 | 2 | 425 | 425 |
| At5g18700sunf32533211 | 846 | 2 | 503 | 89 |
| At5g22300sunf32541569 | 643 | 2 | 410 | 410 |
| At5g21920sunf32531775 | 610 | 2 | 383 | 383 |
| At5g14250sunfQHK18A24_yg_ab1 | 577 | 2 | 374 | 374 |
| At5g22790sunfQHB12O13_yg_ab1 | 558 | 2 | 299 | 0 |
| At3g13530sunfQHB38C17_yg_ab1 | 487 | 2 | 84 | 84 |
| At5g22580sunf32532237 | 462 | 2 | 160 | 0 |
| At2g23540sunf28370478 | 433 | 2 | 86 | 0 |
| At3g01440sunfQHL7G11_yg_ab1 | 411 | 2 | 97 | 95 |
| At1g30380sunfQHM18P23_yg_ab1 | 399 | 2 | 115 | 115 |
| At2g33560sunfQHM13K02_yg_ab1 | 397 | 2 | 92 | 92 |
| At1g20225sunf32537182 | 383 | 2 | 108 | 108 |
| At3g03790sunfQHH6M19_yg_ab1 | 375 | 2 | 74 | 74 |
| At1g17220sunfQHL21N13_yg_ab1 | 365 | 2 | 62 | 62 |
| At1g02640sunfQHB38G03_yg_ab1 | 339 | 2 | 96 | 96 |
| At3g57000sunf32533219 | 323 | 2 | 89 | 0 |
| At3g52140sunfQHF9E07_yg_ab1 | 309 | 2 | 88 | 88 |
| At5g09570sunf32538691 | 299 | 2 | 82 | 82 |
| At5g16270sunf32533888 | 297 | 2 | 79 | 79 |
| At5g05740sunfQHL2P06_yg_ab1 | 297 | 2 | 86 | 86 |
| At1g06690sunfQHE5K07_yg_ab1 | 294 | 2 | 108 | 108 |
| At2g35500sunf32541292 | 282 | 2 | 80 | 80 |
| At1g77550sunf32541543 | 281 | 2 | 92 | 92 |
| At3g10330sunfQHF9F04_yg_ab1 | 271 | 2 | 82 | 82 |
| At4g36440sunfQHK9D01_yg_ab1 | 230 | 2 | 93 | 83 |
